# Circulating Cancer Biomarkers

**DOI:** 10.3390/cancers13040802

**Published:** 2021-02-15

**Authors:** Anna Lokshin, Robert C. Bast, Karin Rodland

**Affiliations:** 1 Departments of Pathology, Medicine, and Obstetrics and Gynecology, University of Pittsburgh Medical Center and University of Pittsburgh Cancer Institute, Pittsburgh, PA 15213, USA; 2 Department of Experimental Therapeutics, University of Texas M.D. Anderson Cancer Center, 1400 Pressler Street, Unit 1439, Houston, TX 77030, USA; rbast@mdanderson.org; 3 Biological Sciences Division, Pacific Northwest National Laboratory, Richland, WA 99354, USA; Karin.Rodland@pnnl.gov; 4 Department of Cell, Developmental, and Cancer Biology, Oregon Health & Science University, Portland, OR 97221, USA

Cancer is among the major public health problems worldwide, representing the leading cause of morbidity and mortality in industrialized countries. In 2020, an estimated 1,806,590 new cases of cancer were diagnosed in the United States and 606,520 people died from the disease [1]. Several major developments have been achieved in cancer treatment, including combination chemotherapy, targeted therapy, immunotherapy, and novel delivery systems, contributing to a decrease in overall cancer death rates. Additionally, changes in lifestyle (smoking) and early detection (mammography, colonoscopy, and PAP smears) have also contributed to a decrease in cancer mortality. Despite all these achievements, the overall cancer death rate in the United States has declined only by 1.4–1.8% since the early 1990s, indicating that additional approaches are needed to further reduce cancer mortality. The approaches with the greatest potential to reduce cancer mortality include the ability to accurately assess an individual’s risk of developing cancer, to detect cancers at early stages when they can be more effectively treated, to distinguish aggressive from nonaggressive cancers, and to detect recurrence and monitor response to therapy (Table 1). Such tests could be based on biomarkers-molecules that indicate the presence of cancer. Cancer biomarkers can represent changes in the genetic, epigenetic, proteomic, glycomic, and metabolomic profile of normal tissues and/or changes in imaging. Each application of cancer biomarkers has specific targets, characteristics, and frequency of testing (Table 1).

Biomarkers can be analyzed in resected tumor tissue, particularly for prognostic and theranostic applications. The FDA has approved the first liquid biopsy companion diagnostic that also uses next-generation sequencing (NGS) technology to identify patients with specific types of mutations of the epidermal growth factor receptor (EGFR) gene in nonsmall cell lung cancer (NSCLC) that will guide treatment decisions.

However, when decisions have to be made in the absence of repeated biopsy and where heterogeneity exists within primary and metastatic sites, noninvasive methods that reflect tumors body-wide should be used. In this case, biomarkers that can be measured in blood or other bodily fluids, e.g., urine, saliva, cyst fluid, ascites, and pleural fluid, could be of great use. Cancer biomarkers can appear in the blood circulation by active secretion or cellular leakage from cancer cells or supporting tissue in the tumor microenvironment. Circulating biomarkers that include proteins and auto-antibodies, nucleic acids (cell-free DNA and RNA), circulating tumor cells, and microvesicles disseminated in the blood stream can serve as a measure of tumor load and metastatic potential, while also providing an opportunity to investigate detailed molecular alterations in a tumor. These biomarkers represent a powerful alternative to taking invasive biopsies of specific organs for molecular analysis, hence the current emphasis on so-called “liquid biopsies” [2]. 

Several requirements must be fulfilled before a cancer biomarker can be approved for clinical use. Biomarker performance is assessed by several parameters, including sensitivity (SN, the proportion of true cancers detected), which is balanced by specificity (SP, the percentage of positive results that are cancer). Combining incidence (frequency of the cancer in the population) with specificity provides the positive predictive value (PPV) necessary for acting on a positive result, while sensitivity combined with incidence provides the negative predictive value (NPV), which provides the rationale for not acting on a negative test result. The optimal requirements for these parameters for any biomarker depend on the purpose of a test and the type of cancer and must take into account the consequences of producing either a false positive or false-negative result. For example, a clinically useful screening biomarker should be able to detect the disease at an early and asymptomatic stage and result in decreased morbidity or increased survival rates. A screening test must be highly specific to minimize false positives and thus the number of unnecessary diagnostic procedures with the associated risk, psychological stress, and cost. In contrast, diagnostic tests in patients suspected of having cancer should have high SN in order not to miss any cancers, but may have a lower SP, as the population subjected to diagnostic tests is at a higher risk of having the disease.

The quest for noninvasive biomarkers suitable for the early detection began in earnest in the 1970s and 1980s when several so-called “cancer antigens” (or CA) were discovered by injecting an extract of a human cancer tissues into animals and testing the animal serum for antibodies against the human antigens present in the extract. The first clinically relevant cancer biomarker identified by this approach in 1965 was cancer embryonic antigen (CEA) in colon cancer tissue extracts [3,4]. This was followed by prostate-specific antigen PSA [5], AFP [6], CA19.9 [7], CA72.4 [8], CA125 [9], and CA 15.3 [10]. Although it was initially felt that these “cancer antigens” were specific to malignant tissue, it soon became apparent that these are by and large surface glycoproteins that are relatively highly abundant in certain tissues, such as kallikrein 2 (PSA), which is restricted to the prostate, and CA125, which is enriched in the Mullerian epithelium of the female reproductive tract as well as in the lung [11,12], and thus they really only reflect changes in the mass of the affected tissue, whether due to malignancy or benign enlargement. These cancer antigens are currently approved by the Food and Drug Administration for clinical use for purposes of prognosis, diagnosis, therapy monitoring, staging, or detection of recurrence. Of note, all of these purposes apply to patients with diagnosed cancer, where the tumor size is substantial. Since then, a large number of circulating cancer-associated biomarkers have been reported in numerous publications. Several of these biomarkers have demonstrated clinically relevant classification power for discriminating cancers from benign controls (differential diagnosis). For example, OVA1^®^ (CA125, transthyretin, apolipprotein A1, beta-2 microglobulin, and transferrin) and ROMA^®^ (CA125+HE4) have been designated as Class II devices by the FDA. They are quantitative serum tests that employ specific algorithms to ascribe a risk score for the presence of ovarian cancer to determine patients with pelvic masses who would benefit from referral to a gynecologic oncologist for their surgery.

As important as the ability to prognosticate, diagnose, stage, and monitor the therapy, and detect the recurrence of a given cancer is, the ability to diagnose the disease early could have the largest impact on mortality. At present, improving methods to screen asymptomatic populations for the presence of early stage cancers is a particularly challenging problem. Cancer is an insidious disease; genetic studies suggest that many cancers lie dormant, developing solely over a decade or more, before clinically significant symptoms appear [13,14]. This growth pattern has serious consequences for patient outcomes; almost without exception, cancers that are detected early, while still organ confined, can be treated with reasonable success by surgical excision combined with a variety of supportive ablative therapies, such as radiation or chemotherapy. Conversely, the prospects following a diagnosis of stage III/IV cancer that has spread even just to regional lymph nodes, are significantly less sanguine. Even highly efficacious initial therapies that produce an initial complete response are transient, with subsequent recurrence and development of resistance all too common, and all too lethal (https://seer.cancer.gov/statistics/ (accessed on 14 February 2021)).

The search for early detection biomarkers is based on the hypothesis that cancer initiation and progression is accompanied by changes in the profile of various molecules over time, either in the emerging malignant cells or the tissue microenvironment, and that at least some of these signature molecules will be shed into blood and other bodily fluids, in sufficient concentrations for detection prior to the onset of clinical symptoms. Unfortunately, with the exception of PSA, no cancer biomarker has been approved for cancer screening and early detection, and even PSA screening is currently controversial due to the high rate of false positives and mixed success in reducing mortality across several large studies [15].

These limitations in the area of screening could be explained by the fact that all cancer biomarkers reported to date were discovered in patients with diagnosed cancer generally bearing large volumes of cancer. For this reason, almost all of the commonly assayed biomarkers measure cancer volume, and consequently, their diagnostic power is limited in small early stage asymptomatic and pre-diagnostic tumors. Thus, by study design, these biomarkers may perform well for cancer diagnosis or for monitoring the effects of therapy, where cases represent advanced cancers. The performance of biomarkers validated in post-diagnostic patients is not guaranteed in pre-diagnostic samples. In fact, recent results in large cohort studies demonstrate that the performance of many cancer biomarkers, which detect disease relatively well among symptomatic patients with diagnosed cancer, drops significantly in patients where samples were collected > 6–12 months before diagnosis [16,17,18,19].

One possible explanation for this consistent failure of biofluid-based protein biomarkers to be validated in large clinical trials, particularly for the most clinically useful application of pre-symptomatic detection, is the high degree of heterogeneity in human populations, so that the range of protein concentrations across individuals may approach or exceed the difference between pre- and post-cancer in the same individual. One approach to avoid this confounding variable is the longitudinal collection of biofluid samples from the same individual, to establish a baseline for that individual. Any departure from that individual’s baseline would signal a physiologically significant change. This approach is incorporated into the ROCA algorithm, which includes CA125 velocity, and has received further support from a recent longitudinal study of head and neck cancer using samples acquired two and four years prior to diagnosis from cases and matched controls [20], respectively.

Thus, new concepts or new technological approaches should be considered. Recently, two promising approaches to cancer detection have been developed based on circulating cell-free DNA. The first test, CancerSeek, is based on the presence of a signature of mutations in cell free DNA. The median sensitivity of CancerSEEK was reported to be 73% for stage II cancers and 43% for stage I cancers at >99% SP. In addition, CancerSEEK localized the cancer to a small number of anatomic sites in a median of 83% of the patients [21]. The second test, GRAIL, evaluates methylation of the most informative regions of the genome and is designed to use its proprietary database and machine-learning algorithms to both detect the presence of cancer and identify the tumor’s tissue of origin. Clinical data have shown the ability of this technology to detect more than 50 cancer types at >99%SP. When looking at the most deadly cancer types, which include bladder, colorectal, esophageal, head and neck, liver, lung, ovarian, pancreatic, gastric lymphoma, and plasma cell neoplasm, the SN was 39% in stage I and 69% in stage II cancers. However, for both tests, the performance was uniform across cancers, being lower for some, including ovarian cancer. The GRAIL test is currently under investigation in a clinical trial using asymptomatic patients randomly recruited from the general population, and interim results indicate an acceptable level of false positives [22]. It was recently estimated that such multicancer early detection tests could result in a substantial reduction in mortality and a significant stage shift in prevalent cancers [23]. The main limitation of both studies is that patients with symptomatic cancers were enrolled. Since both tests analyze cfDNA whose presence is proportionate to the size of tumor, they may be less effective in detecting asymptomatic tumors.

The search for cancer biomarkers has a substantial history, with marked success in the areas of diagnostic, prognostic, and theranostic biomarkers. However, the search for effective noninvasive biomarkers capable of identifying some of the most lethal cancers, such as prostate, ovarian, and pancreatic cancers, prior to the appearance of significant clinical symptoms, has been essentially stalled for the last decade. It is obviously time for a new approach. We suggest the use of a more longitudinal approach, based either on oncogenic changes in circulating DNA (cfDNA or ctcDNA), or on changes over time in protein or other biomarkers in accessible biofluids. The essential implementation of this strategy would involve distinct phases. First, baseline levels of the biomarker of interest would be established for each individual screened, most likely in a routine healthcare setting. The emphasis would be on biomarkers of high sensitivity, even at the cost of specificity, as the objective would be to identify a high-risk population for secondary screening focused on specificity. An increase in the patient’s established baseline level of expression, whether of circulating oncogenic DNA, proteins, or other relevant molecules, would trigger follow-up tests, ideally using an orthogonal measurement. Furthermore, special focus should be given to establishing banks of serial plasma and serum in large enough amounts to conduct DNA, miRNA, and microvesicle analysis. Since circulating biomarkers lack information about location, new improved imaging tests with high specificity, sensitivity, and acceptable costs would be highly desirable, particularly if surgical excision or other ablative technologies were the treatment of choice. Conversely, where imaging is used as a screen, such as spiral CT for lung cancer or mammography for breast cancer, an orthogonal nucleic acid or protein test on biopsy specimens could provide important prognostic information relevant to active surveillance protocols. While there are currently significant technical and logistical challenges to such a multipronged screening strategy, we believe that these challenges can eventually be resolved to provide a significant clinical tool for the early detection and effective treatment of multiple cancers.

## Figures and Tables

**Table 1:** Use of cancer biomarkers.

Condition	Healthy	Healthy/ Asymptomatic	Symptomatic		Diseased		
Purpose of biomarker use	Risk Assessment	Screening/ Early Detection	Differential Diagnosis Treat	Treatment Stratification	Prognosis	Therapy monitoring	Recurrence
Screening targets	Predisposition to developing disease	Identifying individuals with early disease	Detection of cancer vs. benign disease	Detecting targetable mutations	-	Assessing therapy effects	Detecting early recurrence
Useful biomarkers	Mutations, Family History Risk Factors	Biomarkers in bodily fluids Imaging	Biomarkers in bodily fluids Imaging	Genomics	Genomics Biomarkers in bodily fluids	Biomarkers in bodily fluids Imaging	Biomarkers in bodily fluids Imaging
Frequency	Once	Yearly	Once	Once	Once	3–4 weeks	3–4 months
